# Training of pediatric cardiac surgeon in India

**DOI:** 10.4103/0974-2069.43887

**Published:** 2008

**Authors:** Pranava Sinha

**Affiliations:** Department of Cardiovascular Surgery, Children's National Medical Center, 111, Michigan Avenue, NW, Washington-200 10, DC – USA

Sir,

I read with interest, Dr Sharma's thought provoking article on the “Making of a Pediatric Cardiac Surgeon, in India”.[1] The anecdotes described appropriately highlight the continuous evolution and learning by day-to-day experiences in the career of a pediatric cardiac surgeon. I also thank him for subtly raising the sensitive issue of cardiothoracic surgical training in our country.

Recently, the American Board of Thoracic Surgery (ABTS) revised the training requirements for American Residents training in cardiothoracic surgery to a minimum operative requirement of 125 index cases per year.[2] At the same time the Residency Review Committee (RRC) has laid down strict regulations on resident's working hours, not greater than 80 hours per week, which is monitored regularly.[3] All programs have clear-cut job descriptions with multiple feedbacks to ensure adherence to training objectives, which are measured by internal and external auditing systems. The intention behind all this is to maximize clinical training and at the same time safeguarding the residents from unnecessary harassments. At the end of his/her training, an American graduate is expected to be adequately trained to be able to handle the complete spectrum of cases in the specialty. There is a similar system in place in Europe and Australia as well.

On the contrary, the cardiothoracic resident in India still works for endless hours, and apart from his clinical responsibilities, is often expected to do jobs that do not fit into a “doctor’s” job description by any means. In return, the actual clinical training is far from standard and, barring a few exceptional centers, comprises a mere handful of cases as the primary surgeon and the rest as an assistant surgeon. No wonder at the end of residency, he/she is compelled to spend another few years as an apprentice in some foreign country.

Despite globalization, the plight of a resident doctor in India hasn’t changed much. Lack of funds to develop an infrastructure conducive for ideal training is often cited as the prime reason. However, in my opinion, the attitude toward training the residents is the biggest hurdle. It needs to be realized that the resident is not someone to fill in the blanks and take care of the menial jobs that the infrastructural deficiencies have not provided for, but has goals and objectives that have to be met.

Multiple studies have shown no significant difference in results in patients undergoing cardiac surgery between residents operating under supervision and staff surgeons.[4–6] Knowing this, providing a resident with ample operative exposure is the least any trainer can do to fulfill his responsibility. It is imperative that upon completion of training, the graduating resident is competent enough to handle at least routine cardiothoracic cases.

Complex pediatric heart diseases can be much more challenging to fix, and need more refined skills and understanding on the part of the surgeon. Needless to say if the present training of residents does not even make them proficient in managing straightforward adult cardiac surgical cases independently, how can one expect to train good pediatric cardiac surgeons?

This letter was forwarded to Dr. Sharma who is in complete agreement with the views expressed by the Author - Editor
